# Positive regulatory interactions between YAP and Hedgehog signalling in skin homeostasis and BCC development in mouse skin *in vivo*

**DOI:** 10.1371/journal.pone.0183178

**Published:** 2017-08-18

**Authors:** Bassem Akladios, Veronica Mendoza Reinoso, Jason E. Cain, Taopeng Wang, Duncan L. Lambie, D. Neil Watkins, Annemiek Beverdam

**Affiliations:** 1 School of Medical Sciences, UNSW Sydney, New South Wales, Sydney, Australia; 2 Hudson Institute of Medical Research, Clayton, Victoria, Australia; 3 Department of Molecular and Translational Science, Monash University, Clayton, Victoria, Australia; 4 IQ Pathology, Brisbane, Queensland, Australia; 5 The Kinghorn Cancer Centre, Garvan Institute of Medical Research, Darlinghurst, New South Wales, Sydney, Australia; 6 St. Vincent's Clinical School, Faculty of Medicine, UNSW Australia, Darlinghurst, New South Wales, Sydney, Australia; 7 Department of Thoracic Medicine, St Vincent's Hospital, Darlinghurst, New South Wales, Sydney, Australia; 8 The School of Biomedical Sciences, The University of Queensland, Queensland, Brisbane, Australia; University of Queensland Diamantina Institute, AUSTRALIA

## Abstract

Skin is a highly plastic tissue that undergoes tissue turnover throughout life, but also in response to injury. YAP and Hedgehog signalling play a central role in the control of epidermal stem/progenitor cells in the skin during embryonic development, in postnatal tissue homeostasis and in skin carcinogenesis. However, the genetic contexts in which they act to control tissue homeostasis remain mostly unresolved. We provide compelling evidence that epidermal YAP and Hedgehog/GLI2 signalling undergo positive regulatory interactions in the control of normal epidermal homeostasis and in basal cell carcinoma (BCC) development, which in the large majority of cases is caused by aberrant Hedgehog signalling activity. We report increased nuclear YAP and GLI2 activity in the epidermis and BCCs of K14-CreER/Rosa-SmoM2 transgenic mouse skin, accompanied with increased ROCK signalling and ECM remodelling. Furthermore, we found that epidermal YAP activity drives GLI2 nuclear accumulation in the skin of YAP2-5SA-ΔC mice, which depends on epidermal β-catenin activation. Lastly, we found prominent nuclear activity of GLI2, YAP and β-catenin, concomitant with increased ROCK signalling and stromal fibrosis in human BCC. Our work provides novel insights into the molecular mechanisms underlying the interplay between cell signalling events and mechanical force in normal tissue homeostasis *in vivo*, that could potentially be perturbed in BCC development.

## Introduction

Basal cell carcinoma (BCC) is the most common form of human neoplasia and it accounts for more than 70% of non-melanoma skin cancers (NMSC) cases [1]. BCCs originate from the basal keratinocyte layer of the epidermis [2], and are typically caused by aberrant Hedgehog (Hh) signalling activity [2–8].

The Hedgehog (Hh) pathway is involved in the development of every major organ, including the skin [9]. Mammalian Hedgehog signalling occurs via three homologous ligands; Sonic (SHH), Desert (DHH) and Indian (IHH) Hedgehog [10–12], which bind transmembrane protein PTC1, a tumor suppressor, with similar affinity, but display tissue and temporal specific expression patterns [13, 14]. Hedgehog interactions with the transmembrane protein PTC1 relieves the inhibition of Smoothened (SMO) and permits downstream signaling activity, resulting in the activation of the effector protein GLI2. GLI2 then translocates to the nucleus where it regulates the transcription of Hedgehog-pathway target genes including *Gli1* and *Ptc1*, and multiple cell cycle genes, including *N-myc* and *E2F1* transcription factors, all of which contribute to proliferation [15].

SHH signaling also controls epidermal development and homeostasis. SHH produced in the dermal matrix signals to PTC1 in the dermal papilla to activate hair follicle development in the fetal epidermis [16, 17]. Postnatally, Hedgehog signaling in the dermal papilla stimulates bulge stem cells to proliferate and hair follicle down growth during anagen, the growth phase of the hair follicle cycle [18–20]. In addition, *Ptc1* is also expressed in the basal epidermis, and overexpression of *Shh*, or conditional inactivation of *Ptc1* in the basal epidermis or C-terminal truncation of PTC1 result in a severe overgrowth phenotype of the epidermis resembling BCC [21–24]. Furthermore, epidermal SHH has recently been shown to also control papillary fibroblast activity and dermal ECM remodelling [25]. These data demonstrate that Hedgehog signalling controls basal epidermal stem/progenitor cell proliferation both in the epidermal and dermal compartments of the skin. However, the precise regulatory mechanisms of how Hedgehog signalling controls epidermal stem/progenitor proliferation remain unclear.

Hippo/YAP signalling is a master regulator of cell proliferation and organ size [26–29]. YAP is an oncoprotein and transcriptional co-activator, the overexpression and nuclear accumulation of which have been detected in many human cancers [30–36]. Classically, the core Hippo kinase cassette is known to control YAP activity [37, 38]. However, YAP has recently been recognized as a mechanosensor that is activated in response to tissue stiffness irrespective of Hippo kinase pathway activity [39, 40].

YAP plays a pivotal role in epidermal regeneration. It is expressed throughout the epidermis, including in the basal epidermal stem/progenitor cell populations [28, 41, 42]. Overexpression of hyperactive YAP protein mutants in the nuclei of basal keratinocytes drives β-catenin activation and increased basal epidermal cell proliferation rates eventually resulting in epidermal hyperplasia [28, 29, 41, 42]. In addition, transgenic epidermal transplants expressing YAP develop into invasive squamous cell carcinoma (SCC)-like tumour masses in nude mice [41], and YAP expression is strongly upregulated and nuclearly localized in keratinocytes of invasive human non-melanoma skin tumours [42]. These data unequivocally establish that tight regulation of YAP activity is essential for normal epidermal homeostasis, and that aberrant nuclear YAP activity results in tumour development in the epidermis. Nevertheless, the genetic mechanisms controlled by YAP that regulate epidermal stem cell proliferation or cause skin cancer development remain unknown.

Many reports have previously demonstrated crosstalk between the Hedgehog and YAP signalling in control of tissue regeneration and cancer development [34, 43–48]. In this report, we investigated whether YAP and Hedgehog signalling undergo regulatory interactions in control of normal epidermal homeostasis and in skin cancer development. We found increased activity of pathway effectors YAP and GLI2 in the hyperplastic epidermis of mouse models with activated Hedgehog signalling (K14-CreER/Rosa-SmoM2) or activated YAP (YAP2-5SA-ΔC) in the basal epidermis, respectively. Furthermore, we found increased epidermal ROCK signalling, fibroblast activity and dermal fibrosis in the skin of K14-CreER/Rosa-SmoM2 mice. We also found prominent nuclear activity of YAP and β-catenin, and increased ROCK signalling and fibrosis in human BCC. These data strongly support the existence of positive regulatory interactions between YAP, Hedgehog and ROCK mechanosignalling in epidermal homeostasis that may underpin BCC development.

## Materials and methods

### Human sections and mouse experimentation

Animal experimentation and human histological stainings were conducted under protocols approved by the UNSW Australia’s Animal Care and UNSW Human Research Ethics Advisory Panel, and in compliance with the National Health and Medical Research Council ‘Australian code of practice (8^th^ edition, 2013). We have previously described the generation of YAP2-5SA-ΔC transgenic mice [28]. Mouse strains *CtnnB1*^*lox/lox*^ (004152) [49], Rosa-SmoM2 (005130) [50] and K14-CreERT mice (005107) [51] were obtained from the Jackson Laboratories. Conditional *β-catenin* alleles were excised by daily intraperitoneal injection of 75 mg/kg Tamoxifen (TRC) for five consecutive days. Conditional *SmoM2* alleles were excised in postnatal day 30 *K14-CreER/Rosa-SmoM2* transgenic mice by topical application of Tamoxifen (100ul of 200mg/ml in DMSO) for five consecutive days to a 1.5 x 1.5cm patch of shaved skin on the right flank. Mice were euthanized 7–8 weeks later for tissue collection. Genotyping was performed as previously described [28, 50–54].

### Quantitative RNA expression analysis

Full thickness skin biopsies were homogenized in TRIzol^®^ reagent (Life Technologies), and RNA and protein were prepared as recommended by the manufacturer [55]. Quantitative real-time reverse transcriptase–PCR assays were carried out using Fast SYBR^®^ Green Master Mix (Life Technologies 4385612) and Mx3000P qPCR System (Agilent Technologies), and were analysed by the comparative cycle time method, normalizing to *18S* ribosomal RNA levels. Quantitative real-time reverse transcriptase–PCR primers: *Ctgf*-F: 5'- CCCTGCCCTAGCTGCCTACCG-3', *Ctgf*-R: 5'- GCTTCGCAGGGCCTGACCAT-3', *Gli2*-F: 5'-GCAGACTGCACCAAGGAGTA-3', *Gli2*-R: 5'-CGTGGATGTGTTCATTGTTGA-3', *Inhba*-F: 5'- CGATGTCACCCAGCCGGTGC-3', *Inhba*-R: 5'-TGTCTTCCTGGCTGTGCCTGACT-3', *Thbs1*-F: 5'- GCGTTGCCAGGCTCCGAGTT-3', *Thbs1*-R: 5'- GGTGCGCAGGCCCTTCAGTT-3', *18S*-F: 5’-GATCCATTGGAGGGCAAGTCT-3’ and *18S-R*: 5’-CCAAGATCCAACTACGAGCTTTTT-3’.

### Tissue processing and histological staining

Full thickness skin biopsies were processed for paraffin sectioning and histology staining using routine methods. Antigen retrieval was performed using 10mM sodium citrate buffer (pH 6.0) and a Milestone RHS-1 Microwave at 110°C for 5 minutes. Sections were immunostained using routine methods, and confocal images were captured using an Olympus FV1200 laser scanning confocal microscope. Immuno-signal intensity was quantified in a semi-automated fashion using ImageJ software. Immunohistochemical staining was performed on paraffin sections using DAB enhanced liquid substrate system (Sigma D3939) according to manufacturer’s instructions (Sigma-Aldrich). Primary and secondary antibodies used for immunofluorescence and immunohistochemical stainings are listed in S1 Table.

### Acquisition and analysis of SHG data from collagen

SHG signal from histological samples was captured using a 20x 1.0 NA objective on an upright fixed-stage two-photon laser scanning microscope system (Zeiss) and analyzed as previously described [56].

### Statistical analysis

Unless indicated, statistical significance was determined by Student’s unpaired *t*-tests. Error bars represent mean ± SEM. Asterisks indicate statistical significance, where *P* < 0.05 was used as significance cut-off.

## Results

### YAP activation in the BCC skin of K14-CreER/Rosa-SmoM2 transgenic mice

Cross-regulatory interactions between YAP and the SHH signalling pathway control stem cell proliferation and tissue homeostasis [34, 43–48]. To investigate if regulatory interactions between these pathways also exist in epidermal homeostasis, we first set out to investigate YAP activity levels in the skin of K14-CreER/Rosa-SmoM2 transgenic mice, which express a constitutively active Smoothened mutant protein SmoM2 in the basal keratinocytes upon tamoxifen treatment, resulting in ligand-independent activation of Hedgehog signalling [50]. The skin of mice expressing SmoM2 in the epidermis displays epidermal hyperplasia and BCC-like tumours [57, 58].

**Fig 1:**
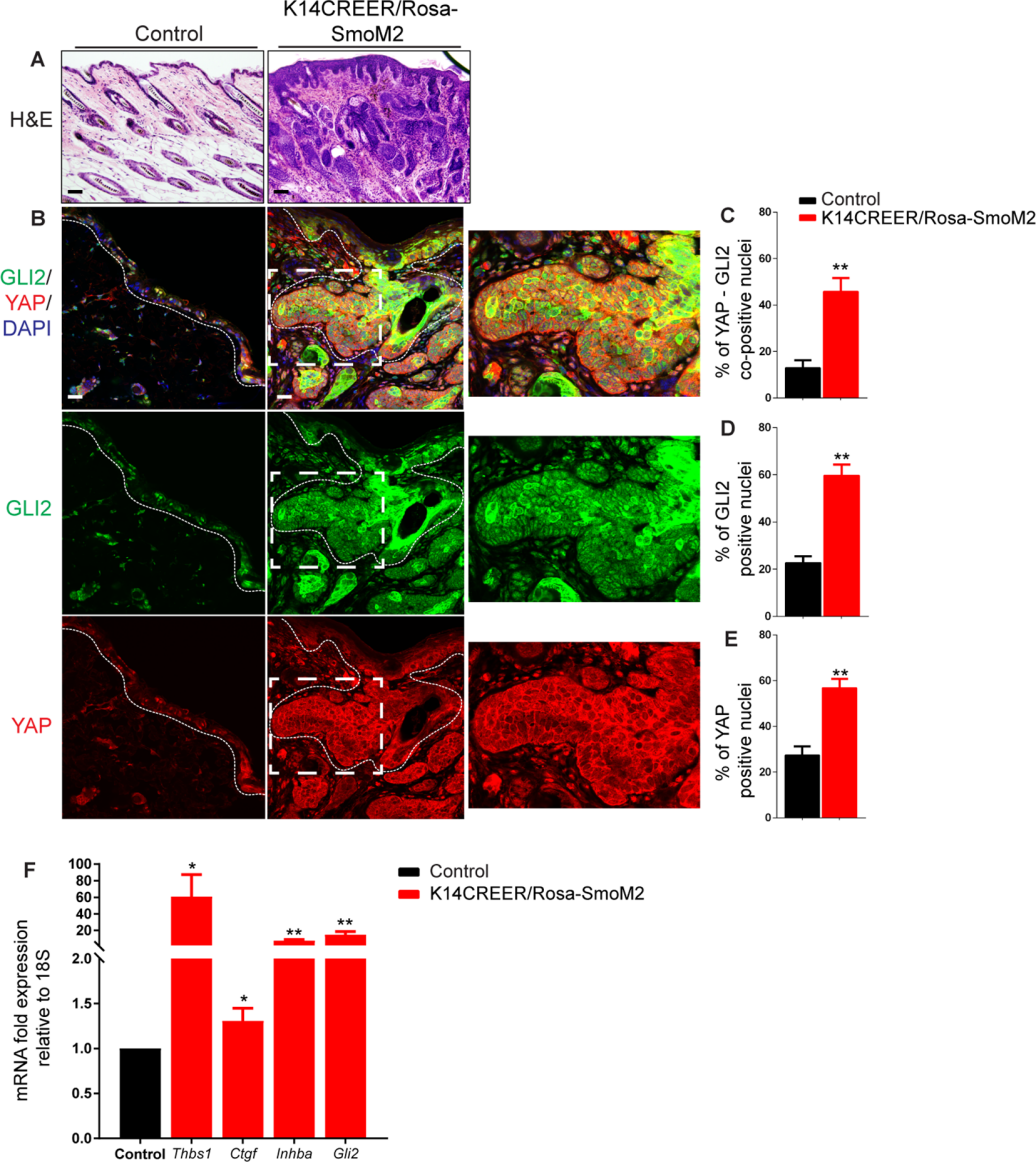
YAP activation in the skin of K14-CreER/Rosa-SmoM2 transgenic mice. H&E histological staining (A) and Immunofluorescence (B) staining of dorsal skin sections of tamoxifen- and vehicle-treated K14-CreER/Rosa-SmoM2 transgenic mice detecting GLI2 and YAP. Quantification of % GLI2-YAP co-positive (C), % GLI2 positive (D) and % YAP positive nuclei (E). (F) qPCR quantification of mRNA levels of *Thbs*, *Ctgf*, *Inhba* and *Gli2* genes relative to *18S* in lysates extracted from the dorsal skin of tamoxifen (control) and vehicle-treated K14-CreER/Rosa-SmoM2 transgenic mice. Basement membranes are demarcated with dashed lines. DAPI, 4, 6-diamidino-2-phenylindole. Scale bars = 20 μm.

K14-CreER/Rosa-SmoM2 transgenic littermate mice were topically treated with tamoxifen or vehicle, and euthanized 7–8 weeks later. The skin of tamoxifen-treated K14-CreER/Rosa-SmoM2 mice displayed hyperplasia and signs of BCC development (Fig 1A), as previously reported [58]. To assess Hedgehog signalling activity, we performed immunofluorescence assays to detect GLI2, the key mediator of SHH responses in skin [59]. These assays revealed an increased percentage of GLI2 positive nuclei both in the epidermis and BCC tumour masses of K14-CreER/Rosa-SmoM2 mouse skin (Fig 1B and 1D; P < 0.01, *N* = 3), confirming increased Hedgehog signalling activity. This was accompanied by a significant increased percentage of YAP positive nuclei within the extending BCC masses of tamoxifen-treated compared to vehicle-treated K14-CreER/Rosa-SmoM2 skin (Fig 1B and 1E; P < 0.01, *N* = 3). Furthermore, the percentage of nuclei co-positive for YAP and GLI2 was also increased in the K14-CreER/Rosa-SmoM2 epidermis (Fig 1B and 1C; P < 0.01, *N* = 3). In addition, quantitative real time PCR assays showed increased expression of YAP direct target genes *Thbs*, *Ctgf*, *Inhba* and *Gli2* [27, 34, 60, 61] in K14-CreER/Rosa-SmoM2 skin (Fig 1F). These findings show that YAP transcriptional activity is increased in the epidermis of K14-CreER/Rosa-SmoM2 mice, and support the hypothesis that Hedgehog signaling positively regulates YAP activity in normal epidermal regeneration and in BCC development.

### Activated ROCK-signalling and dermal fibrosis in the skin of K14-CreER/Rosa-SmoM2 transgenic mice

A recent study revealed that epidermal Sonic Hedgehog (SHH) controls dermal fibroblast activity and dermal composition [25]. Furthermore, ROCK, the effector of the RhoA GTPase, also plays a key role in tissue homeostasis through controlling mechanoreciprocity, including in the skin, and increased ROCK signalling and stromal stiffness are hallmarks of tumour progression [56, 62, 63]. Therefore, we next investigated stromal composition and ROCK signalling activity in the hyperplastic skin and BCCs of K14-CreER/Rosa-SmoM2 mice.

**Fig 2:**
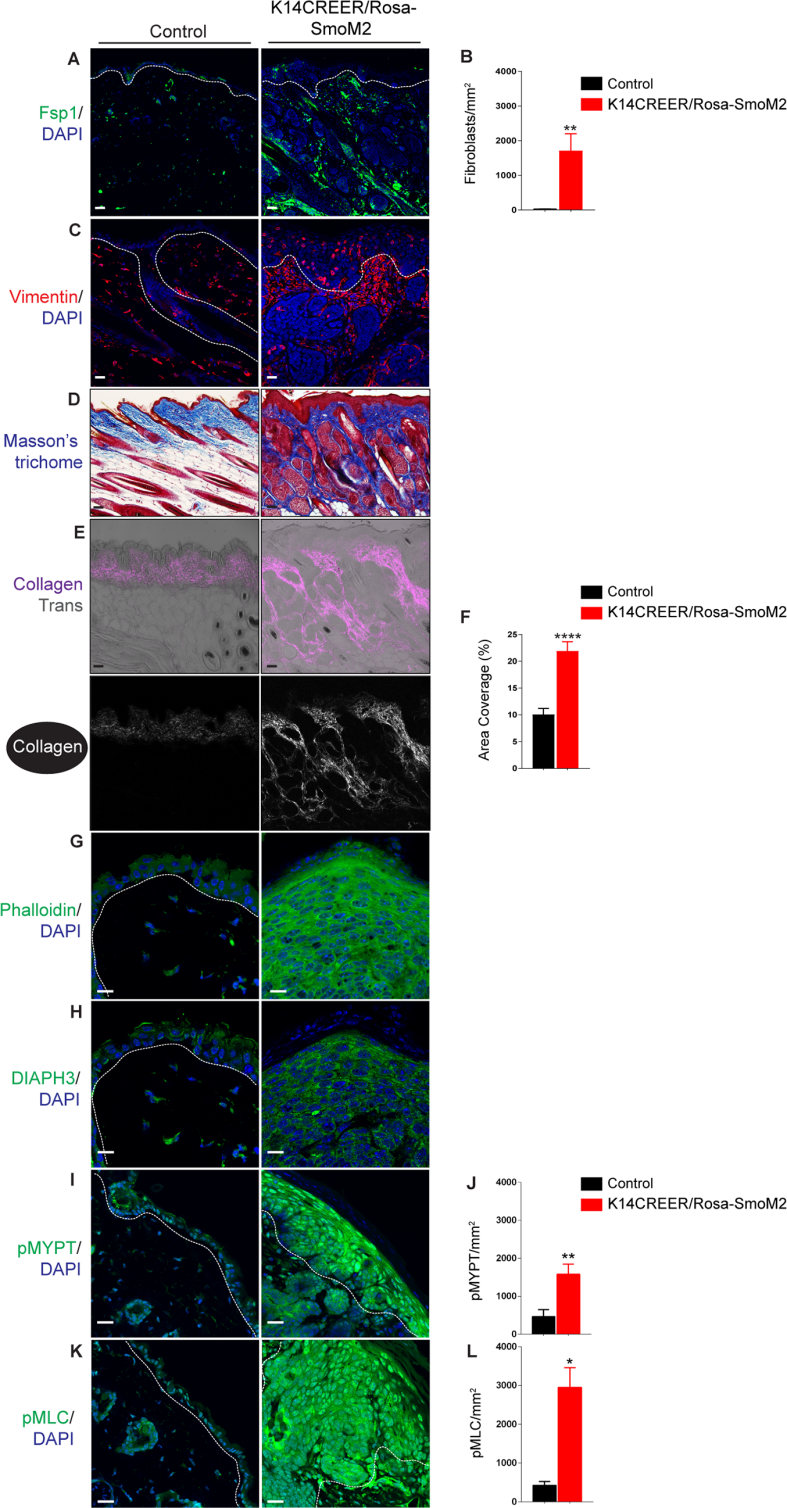
Activated ROCK-signalling, increased dermal fibroblast numbers, and dermal fibrosis in the skin of K14-CreER/Rosa-SmoM2 transgenic mice. (A-G) Immunofluorescence staining and area coverage analysis of dorsal skin tamoxifen- and vehicle-treated K14-CreER/Rosa-SmoM2 transgenic littermate mice detecting Fsp1 (A & B) and Vimentin (C), Phalloidin (G), DIAPH3 (H), Thr696-phosphorylated MYPT1 (I & J), Thr18/Ser19-phosphorylated MLC2 (K & L). (D) Masson’s trichrome histological staining of sections through the dorsal neck skin of tamoxifen- and vehicle-treated K14-CreER/Rosa-SmoM2 mice. (E & J) Dual two-photon SHG and monochromatic transmission (Trans; grayscale in merge) images showing collagen (white in single channel, magenta in merged) in tamoxifen- and vehicle-treated K14-CreER/Rosa-SmoM2 skin sections. Area coverage analysis (5 fields/sample from three mice per genotype) of SHG is quantified. Basement membranes and hair follicles are demarcated with dashed lines. DAPI, 4, 6-diamidino-2-phenylindole. Scale bars = 20 μm.

Immunostaining assays and area coverage analysis detected a significant increase in expression of S100a4/fibroblast specific protein 1 (Fsp1) (Fig 2A & 2B; P < 0.01, *N* = 12) and Vimentin expression (Fig 2C) in the dermis of tamoxifen-treated K14-CreER/Rosa-SmoM2 mice. This was concomitant with a significant increase in dermal collagen density compared vehicle-treated mice, as shown by Masson’s trichrome staining (Fig 2D), and by second harmonic generation analysis (SHG) (Fig 2E & 2F; P < 0.0001, *N* = 15). These data are in line with previously reported [25]. Furthermore, the keratinocytes of the K14-CreER/Rosa-SmoM2 epidermis displayed high levels of actin (Fig 2G) and DIAPH3 expression (Fig 2H), suggesting increased RhoA/ROCK signalling and actin remodelling. This was confirmed by increased phosphorylation of both ROCK substrate proteins the myosin-binding subunit of the Mlc Phosphatase (MYPT1 phosphorylated at Thr696) (Fig 2I & 2J; P < 0.01, *N = 3*) and myosin regulatory light chains (MLC2 phosphorylated at Thr18/Ser19) (Fig 2K & 2L; P < 0.05, *N = 3*) [64] in the epidermis of tamoxifen-treated K14-CreER/Rosa-SmoM2 mice. Taken together, this suggests that epidermal Hedgehog signalling activates ROCK signalling within keratinocytes, and dermal fibroblast activity and matrix remodelling in the mouse skin *in vivo*.

### GLI2 activation in the skin of YAP2-5SA-ΔC transgenic mice

To investigate if epidermal YAP activation also modulates Hedgehog signalling activity similar to previously reported in other biological contexts [46, 48], we investigated GLI2 activation in the skin of the YAP2-5SA-ΔC mouse line. The YAP2-5SA-ΔC mouse line is a viable and fertile transgenic mouse line that expresses a mildly activated YAP protein mutant YAP2-5SA-ΔC in the nuclei of basal keratinocytes, and displays epidermal hyperplasia due to increased epidermal β-catenin activity and epidermal stem/progenitor cell proliferation [28, 29].

**Fig 3:**
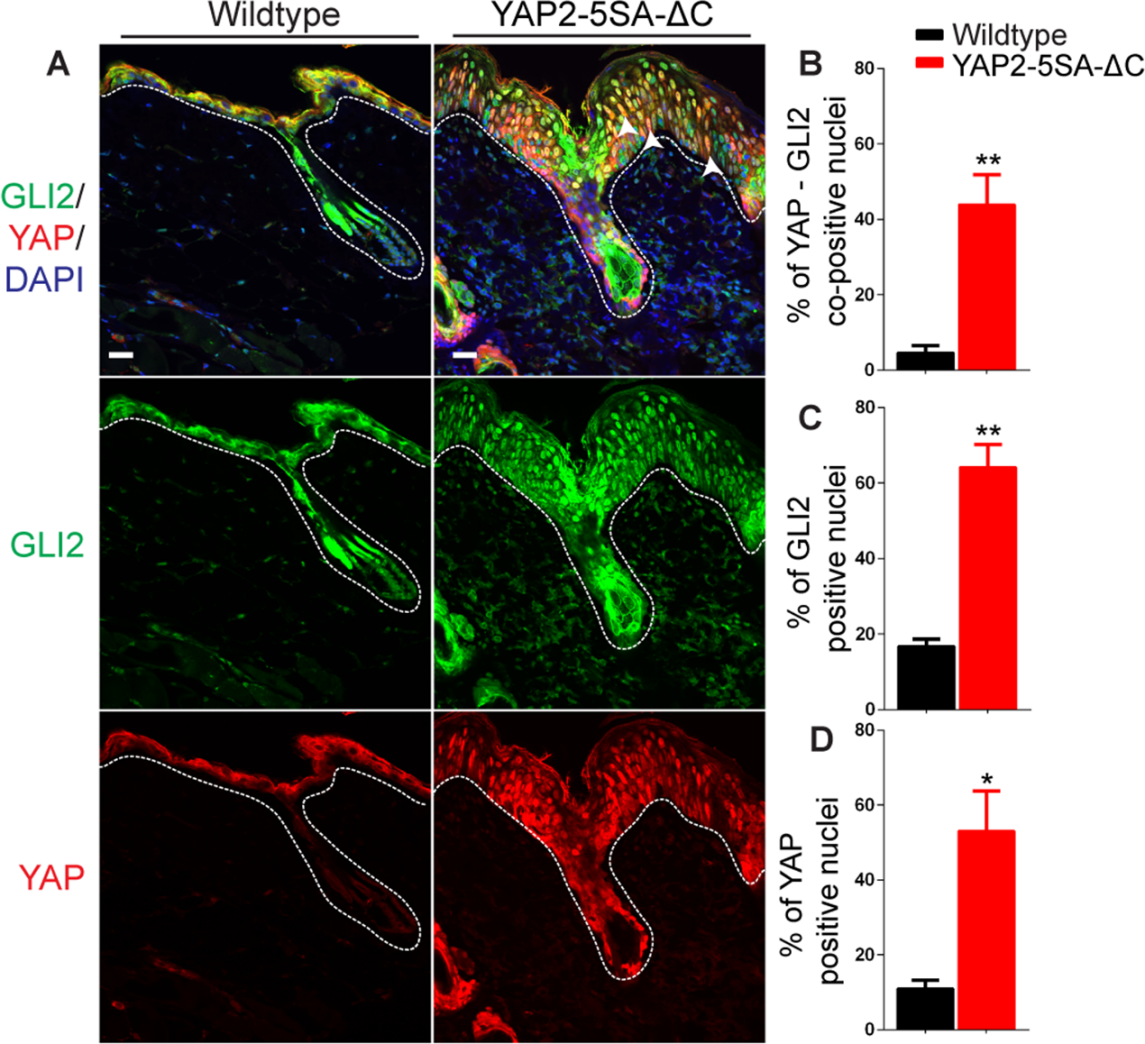
GLI2 activation in the skin of YAP2-5SA-ΔC transgenic mice. (A) Immunofluorescence staining of dorsal skin sections of YAP2-5SA-ΔC transgenic and wildtype mice detecting GLI2 (green) and YAP (red). Quantification of % YAP-GLI2 co-positive (arrowheads—B), % GLI2 positive (C) and % YAP (D) positive nuclei in the skin sections of YAP2-5SA-ΔC transgenic and wildtype mice. Basement membranes are demarcated with dashed lines. DAPI, 4, 6-diamidino-2-phenylindole. Scale bars = 20 μm.

Immunofluorescence staining revealed relatively high levels of nuclear YAP in the hyperplastic epidermis of YAP2-5SA-ΔC skin (Fig 3A & 3D, P < 0.05, *N* = 3), as we previously reported [29]. In addition, YAP2-5SA-ΔC keratinocytes showed a significantly increased percentage of GLI2 positive nuclei *vs*. total nuclei (Fig 3C, P < 0.01; *N* = 3), and an increased percentage of YAP-GLI2 co-positive nuclei (arrowheads-Fig 3A & 3B, P < 0.05; *N* = 3). Altogether, this data shows that YAP activation in basal keratinocytes promotes nuclear localization of GLI2 in the epidermis.

### β-catenin activity mediates GLI2 activation in skin of YAP2-5SA-ΔC mice

We next wanted to better understand the underlying mechanism of GLI2 activation in the epidermis of YAP2-5SA-ΔC mice. A recent report showed that epidermal β-catenin activates epidermal SHH to induce changes in the underlying dermal compartment that involve promoting fibroblasts proliferation [25, 65]. Furthermore, we established that epidermal YAP drives β-catenin activation [29]. Therefore, we next set out to understand if GLI2 activation in YAP2-5SA-ΔC skin depended on increased epidermal β-catenin activity.

**Fig 4:**
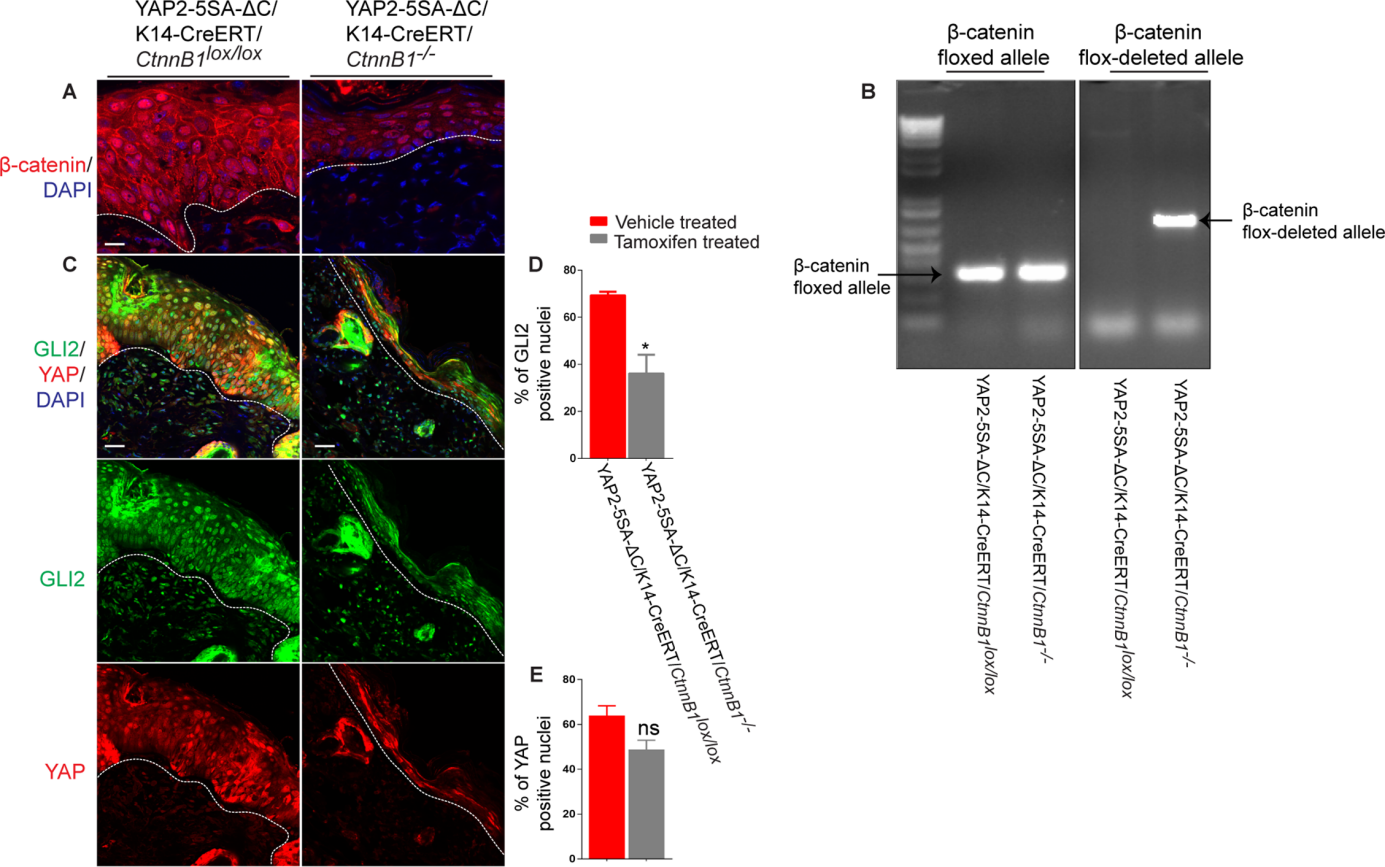
β-catenin activity mediates GLI2 activation in skin of YAP2-5SA-ΔC mice. Immunofluorescence stainings of dorsal neck skin sections of P50 of YAP2-5SA-ΔC/K14-creERT/*CtnnB1*^*lox/lox*^ and YAP2-5SA-ΔC/K14-creERT/*CtnnB1*^*-/-*^ littermate mice detecting β-catenin (A, red), and GLI2 (C, green), and YAP (C, red). (B) Genotypic characterization of P50 YAP2-5SA-ΔC/K14-creERT/*CtnnB1*^*lox/lox*^ and YAP2-5SA-ΔC/K14-creERT/*CtnnB1*^*-/-*^ mutant littermate mice. Quantification of % GLI2 (D) and YAP (E) positive nuclei in the skin sections of P50 of YAP2-5SA-ΔC/K14-creERT/*CtnnB1*^*lox/lox*^ and YAP2-5SA-ΔC/K14-creERT/*CtnnB1*^*-/-*^ littermate mice. Basement membranes are demarcated with dashed lines. DAPI, 4, 6-diamidino-2-phenylindole. Scale bars = 20 μm.

We generated YAP2-5SA-ΔC/K14-creERT/*CtnnB1*^lox/lox^ mice, and studied epidermal GLI2 activation in response to epidermal β-catenin inactivation by tamoxifen treatment. Correct inactivation of the conditional *CtnnB1* allele was confirmed by PCR genotyping (Fig 4B), and by the reduced pan-β-catenin expression levels in the relatively thin epidermis of YAP2-5SA-ΔC/K14-creERT/*CtnnB*^*-/-*^ mice (Fig 4A), in line with our previous observations [29]. We found that the percentages of YAP-positive keratinocyte nuclei in the epidermis were similar in YAP2-5SA-ΔC/K14-creERT/*CtnnB1*^-/-^ and YAP2-5SA-ΔC/K14-creERT/*CtnnB1*^*lox/lox*^ skin (Fig 4E), showing that expression of the transgene product did not depend on epidermal β-catenin activity. Furthermore, we detected a significant decrease in the percentage of nuclear GLI2 in the YAP2-5SA-ΔC/K14-creERT/*CtnnB1*^-/-^ epidermis relative to YAP2-5SA-ΔC/K14-creERT/*CtnnB1*^*lox/lox*^ skin (Fig 4C and 4D, P < 0.05, *N* = 3), to levels more similar as detected in wildtype skin (Fig 2C). These data suggest that YAP activity in basal keratinocytes promotes epidermal GLI2 activation through β-catenin activation.

### Human basal cell carcinomas (BCCs) exhibit nuclear YAP and β-catenin in association with ROCK signalling activation and increased ECM collagen deposition

**Fig 5:**
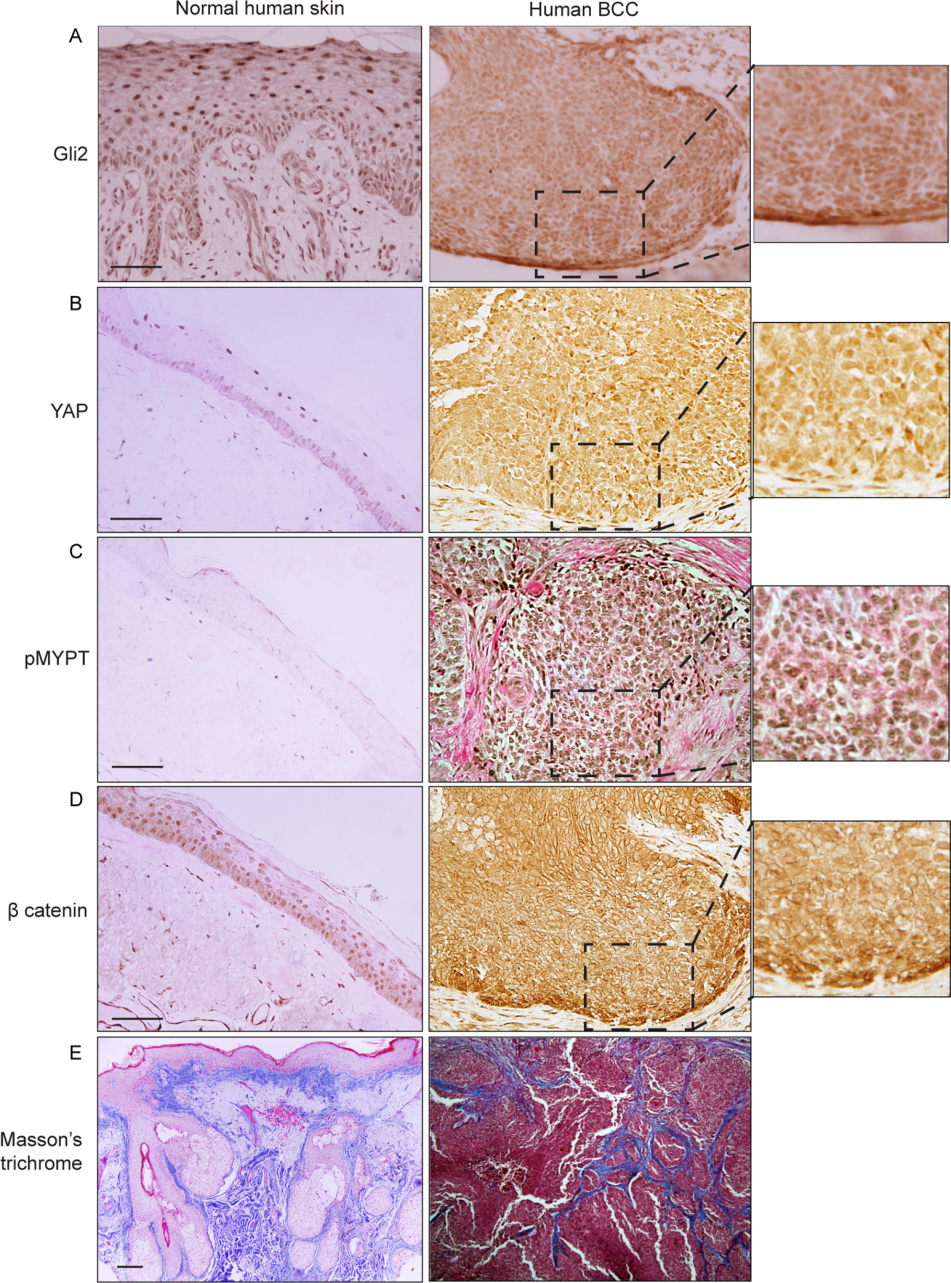
Human BCCs exhibit nuclear YAP and β-catenin in association with ROCK signalling activation and increased ECM collagen deposition. Representative images of immunohistochemical staining (brown) of Gli2 (A), YAP (B), Thr696-phosphorylated MYPT (C) and β-catenin (D) in normal and human BCCs skin samples. (E) Masson’s trichrome histological staining. IHC, Immunohistochemistry. Scale bars = 20 **μ**m.

We found evidence supporting the existence of positive reciprocal regulatory interactions between YAP and Hedgehog signalling in epidermal homeostasis, and we established that Hedgehog signalling also promoted ROCK signalling, dermal fibroblast activity and fibrosis. We next wanted to investigate if these regulatory interactions between Hedgehog, YAP, β-catenin, and ROCK signalling could play a role in the etiology of human BCCs. We investigated a panel of 7 human BCCs, and detected strong nuclear GLI2, YAP and β-catenin expression, concomitant with increased phosphorylation of ROCK-substrate MYPT in cancer keratinocytes of all BCC patients (Fig 5A–5E and S1 Fig) compared to in wildtype skin. In addition, we noted increased collagen deposition in the stroma surrounding all BCCs (Fig 5E and S1 Fig). These findings support the hypothesis that in human BCCs, YAP and SHH signalling activation may indeed be closely linked to the concomitant activation of ROCK-dependent mechanosignalling events, dermal fibrosis and β-catenin activation.

## Discussion

**Fig 6:**
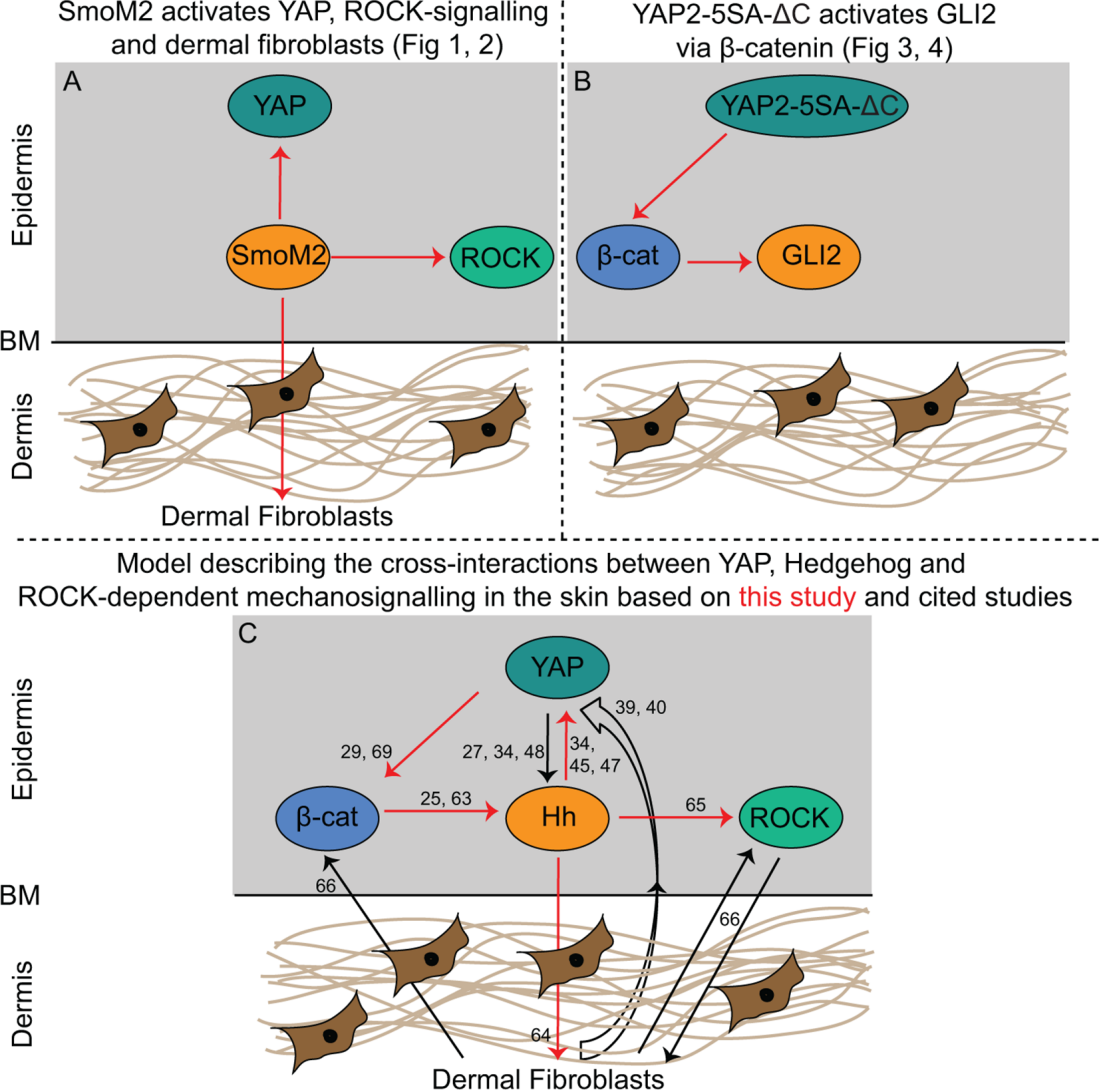
A model outlining the cross-regulatory interactions between epidermal YAP, ROCK, β-catenin and Hh signalling. (A) Epidermal SmoM2 activates YAP, ROCK signalling and dermal fibroblasts in the dorsal skin of K14-CreER/Rosa-SmoM2 transgenic mice (based on Figs 1 and 2). (B) Epidermal YAP activates GLI2 mediated by β-catenin activation in the dorsal skin of YAP2-5SA-ΔC transgenic mice (based on Figs 3 and 4). (C) A model outlining the proposed regulatory interactions between epidermal YAP, Hedgehog and ROCK-dependent mechanosignalling to balance skin regeneration based on our findings (red arrows) and on cited studies [25, 27, 29, 34, 39, 40, 45, 47, 48, 63, 64, 65, 66, 69].

Here we investigated the existence of cross-regulatory interactions between YAP and Hedgehog signalling in the control of epidermal homeostasis. We report increased nuclear GLI2 and YAP in the epidermis and BCCs of K14-CreER/Rosa-SmoM2 transgenic mouse skin and increased expression of YAP direct target genes *Ctgf*, *Gli2*, *Inhba* and *Thbs1*, accompanied by increased ROCK signalling and ECM remodelling (red arrows in Fig 6A). Furthermore, we found that epidermal YAP activity drives GLI2 nuclear accumulation, which depends on epidermal β-catenin activation (red arrows in Fig 6B). Lastly, we found increased nuclear activity of GLI2, YAP and β-catenin, concomitant with increased ROCK signalling and fibrosis, in human BCC. Together, these data are supportive of the existence of positive regulatory interactions between YAP and Hedgehog signalling in control of epidermal homeostasis and in BCC development.

The molecular basis of how Hedgehog signalling may activate YAP in the epidermis of K14-CreER/Rosa-SmoM2 transgenic mice remains unknown. Conceivably, Hedgehog signalling may promote YAP expression levels and activity analogous to in liver regeneration, medulloblastoma, osteosarcoma and neural progenitor cells [34, 45, 47]. Conversely, we observed increased collagen content in the dermis of tamoxifen-treated K14-CreER/Rosa-SmoM2 mice, which may be caused by increased TGFβ signalling in response to SmoM2 in line with previously reported [66]. Therefore, the mechanosensor YAP may also be activated indirectly in response to the increased dermal stiffness [39, 40] due to epidermal Hedgehog signalling activity (Fig 6C).

Our data also show increased ROCK activity, fibroblast numbers and fibrosis in the skin of K14-CreER/Rosa-SmoM2 mice. This may be mediated by increased TGFβ signalling in response to SmoM2, as previously reported [66]. Conversely, Smo was recently reported to directly couple to heterotrimeric G_i_ proteins to activate ROCK signalling, cytoskeletal changes, and fibroblast activity through a GLI2-independent ‘non-canonical’ Hedgehog signalling pathway [67]. Therefore, epithelial SmoM2 overexpression in K14-CreER/Rosa-SmoM2 mice may also activate epidermal ROCK signalling cell-autonomously through G_i_ proteins (Fig 6C) in line with this study [67]. Conversely, papillary fibroblast activation and dermal remodelling in the K14-CreER/Rosa-SmoM2 dermis may indirectly activate ROCK signalling through mechanoreciprocity between the skin layers as previously reported [68]. These data are consistent with the existence of ROCK-dependent mechanoreciprocity in the skin in response to epidermal Hedgehog signalling activity.

Furthermore, we found increased nuclear GLI2 in the epidermis of YAP2-5SA-ΔC mice, suggesting that YAP may also positively regulate Hedgehog signalling in the epidermis, analogous to in other biological contexts [34, 48]. However, we have not been able to firmly establish this, as our quantitative real time PCR experiments did not detect increased expression of Hedgehog pathway target gene *Ptc1* or *Gli1* in the RNA extracted from skin biopsies of YAP2-5SA-ΔC *vs*. wildtype mice (data not shown), an effect that may have been masked by the transcriptomes of other cell types present in these skin biopsies. Nevertheless, we did establish that the positive regulatory interaction between YAP and GLI2 depends on epidermal β-catenin. We previously reported that YAP activity in basal keratinocytes drives β-catenin activation to promote epidermal proliferation [29]. Therefore, GLI2 may be activated in response to increased β-catenin transcriptional activity driven by epidermal YAP activity. Interestingly, a previous report showed TCF/LEF binding sites in the human GLI2 promoter [69], suggesting that this positive regulatory interaction may take place at the transcriptional level.

Interestingly however, we also detected a strong increase in keratinocyte nuclei positive for both GLI2 and YAP (Fig 1C and Fig 3B), signifying that a cell-autonomous mechanism may also underlie this interaction (Fig 6C). GLI2 is indeed a reported direct YAP/TEAD target gene [27], so perhaps the YAP/TEAD transcriptional complex promotes GLI2 transcription in the control of epidermal stem/progenitor cell proliferation, in line with reported for the regulation of cerebellar neural progenitor cell proliferation [34, 48]. An alternative mechanism for which there is no published precedence is that YAP/TEAD may form a transcriptional complex with GLI2 to promote transcription of common target genes to activate epidermal stem/progenitor cells.

Altogether, based on our and previously published work, a picture emerges of an intricate reciprocal mechanosignalling network consisting of the YAP, Hedgehog and β-catenin signalling pathways and ROCK mechanoreciprocity that all regulate each other’s activity cell autonomously or non-autonomously via dermal remodelling, to balance normal skin regeneration (Fig 6C). Therefore, our findings have clinical implications and carry a promise for developing new therapeutic approaches for treating human BCCs.

## Supporting information

S1 Fig BCCs of 7 human patients exhibit nuclear YAP and β-catenin in association with ROCK signalling activation and increased ECM collagen deposition. Representative images of H&E and Masson Trichrome stained sections, and immunohistochemical staining (brown) of Gli2, YAP, Thr696-phosphorylated MYPT and β-catenin of normal and human BCCs skin samples. Scale bars = 20 **μ**m.
(TIF)

S1 Table List of primary and secondary antibodies. (DOCX)
