# Supplementary figures and images for: Positive regulatory interactions between YAP and Hedgehog signalling in skin homeostasis and BCC development in mouse skin in vivo
Source: PLoS One. 2017 Aug 18;12(8):e0183178. doi: 10.1371/journal.pone.0183178 (PMC5562304; doi:10.1371/journal.pone.0183178)

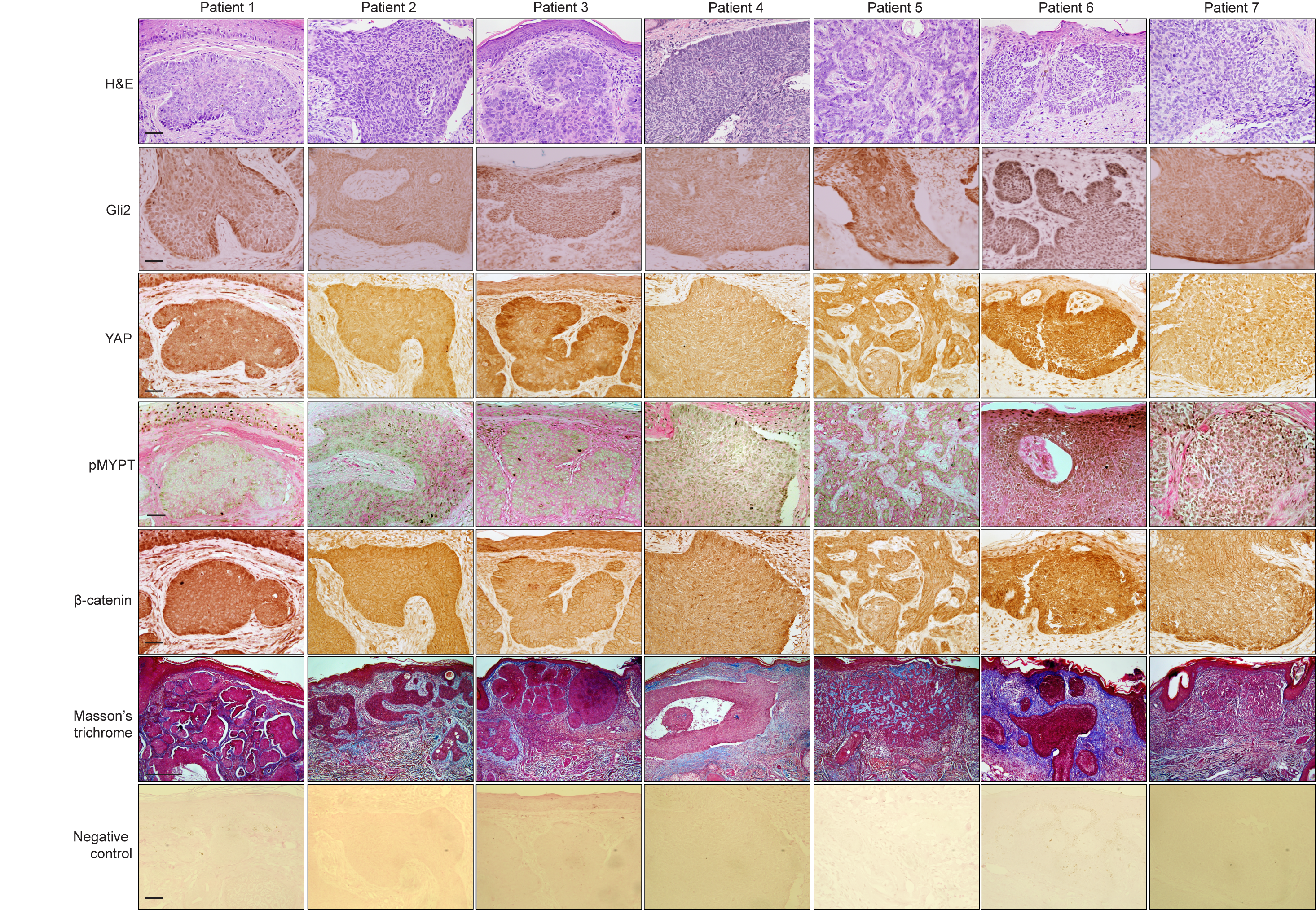

Supplement: S1 Fig — Representative images of H&E and Masson Trichrome stained sections, and immunohistochemical staining (brown) of Gli2, YAP, Thr696-phosphorylated MYPT and β-catenin of normal and human BCCs skin samples. Scale bars = 20 μm. (TIF) [file pone.0183178.s001.tif]
