# Supplementary material for: Positive regulatory interactions between YAP and Hedgehog signalling in skin homeostasis and BCC development in mouse skin in vivo
Source: PLoS One. 2017 Aug 18;12(8):e0183178. doi: 10.1371/journal.pone.0183178 (PMC5562304; doi:10.1371/journal.pone.0183178)
Supplement: S1 Table — (DOCX) [file pone.0183178.s002.docx]

**S1 Table.** List of used primary and secondary antibodies.

| Antibody | Source | Product# | Host | Dilution  IF IHC | |
| --- | --- | --- | --- | --- | --- |
| Active-β-catenin (Clone 8E7) | Merck Millipore | 05-665 | Mouse | 1:200 |  |
| β-catenin | Abcam | ab6302 | Rabbit | 1:200 | 1:200 |
| CyclinD1 | Epitomics/Abcam | ab134175 | Rabbit | 1:200 |  |
| DIAPH3 | Sigma Aldrich | HPA032152 | Rabbit | 1:200 |  |
| Fsp1/S100A4 | DSHB | CPTC-S100A4 | Mouse | 1:50 |  |
| Gli2 | Abcam | Ab7195 | Rabbit | 1:200 |  |
| pMlc2 (Thr18/Ser19) | Cell Signaling | 3674 | Rabbit | 1:50 |  |
| pMYPT (Thr696) | Merck Millipore | ABS45 | Rabbit | 1:50 | 1:50 |
| Vimentin | Cell Signaling | 5741 | Rabbit | 1:200 |  |
| YAP | Cell Signaling | 4912 | Rabbit | 1:200 | 1:200 |
| Atto Phalloidin 488 | Atto-Tec | AD 488-81 |  | 1:200 |  |
| Alexa 594-anti rabbit | Molecular Probes | A-21207 | Donkey | 1:200 |  |
| Alexa 488- anti mouse | Molecular Probes | A-11001 | Goat | 1:200 |  |
| Biotinylated anti rabbit IgG (H+L) | Vector | BA-1000 | Goat |  | 1:200 |
